# Diagnosing Necrotizing Fasciitis Using Procalcitonin and a Laboratory Risk Indicator: Brief Overview

**DOI:** 10.7759/cureus.2754

**Published:** 2018-06-07

**Authors:** Ahsan Zil-E-Ali, Muniba Fayyaz, Arooj Fatima, Zubair Ahmed

**Affiliations:** 1 General Surgery, Fatima Memorial Hospital, Lahore, PAK; 2 Department of Internal Medicine, Fatima Memorial Hospital, Lahore, PAK; 3 Medical Student-3, FMH College of Medicine & Dentistry, Lahore, PAK

**Keywords:** laboratory testing, non-invasive management, necrotizing fasciitis

## Abstract

Necrotizing fasciitis is a progressive inflammatory disease that requires an early diagnosis to avoid limb salvage and other deadly manifestations. The current protocol is the microbiological and histopathological sampling of the tissue. Once the diagnosis is made, it should be managed with antimicrobial therapy, debridement, and surgical interventions. Such interventions can be invasive and increase the time to treat, which may increase morbidity. Our article discusses procalcitonin, C-reactive protein, and other markers, such as "pain out of proportion,” lactate, creatinine, and creatine kinase, to make a quicker diagnosis before proceeding with invasive procedures. We discussed a similar non-invasive approach called the "Laboratory Risk Indicator for Necrotizing Fasciitis" scoring system that can aid in the early diagnosis of necrotizing fasciitis, which can prompt rapid empiric therapy, reducing the chances of morbidity. This scoring system comprises C-reactive protein, white blood cell count, hemoglobin level, creatinine, sodium, and glucose. Such non-invasive, bedside, and quick tests can help in reducing the time required to make the diagnosis and can affect the course of the disease, hence, improving patient outcomes.

## Introduction and background

Necrotizing fasciitis (NF) is a severe and life-threatening soft tissue infection characterized by progressive necrosis of the fascia and subcutaneous tissue occurring along the fascial planes [1]. It is more prevalent among those having diabetes mellitus, the immunodeficient status, malnutrition, and illicit drug usage. The mortality can reach up to 100% if not diagnosed and treated [2]. A patient’s clinical characteristics, surgical exploration, microbiological and histopathological analysis of soft tissue are the gold standard for the diagnosis of NF [1]. These tests can be invasive and time-consuming and delay the treatment, increasing the chances of mortality. This article mentions the benefits of using non-invasive tests, such as procalcitonin (PCT), Laboratory Risk Indicator for Necrotizing Fasciitis (LRINEC) scoring, and other non-invasive serological markers of inflammation that can aid in the diagnosis of NF in its early stages for earlier treatment and better outcomes.

## Review

Use of PCT and LRINEC scoring in diagnosing necrotizing fasciitis

PCT is a precursor of calcitonin; a hormone involved in the homeostasis of calcium. Its normal level in the serum is as low as 0.01 microgram per liter. During infections of bacterial origin and local sepsis, the levels are raised and it is released by the lungs and intestines, acting as a proinflammatory stimulus. In these conditions, it can rise above its normal value, thus, it can serve as a clinical tool in the early detection of these conditions [3-4]. It predicts that the early eradication of the infectious focus can impact the treatment of disease and the recommendation is for it to be checked two days after an operation.

There have been multiple studies that have concluded that elevated levels of PCT are associated with the complicated course of an underlying disease, which can lead to fatality. This determines that raised PCT in the blood can represent severe ongoing tissue damage and, thus, a worsening of the underlying necrotizing fasciitis. In addition, a study by Friederichs et al. illustrated the clinical signs and course of sepsis in necrotizing soft tissue infections (NSTI). This study mentioned that the value of PCT post-operatively, on days one and two, can be a significant indicator of successful surgical eradication [5]. This points to the fact that PCT is not only a diagnostic marker but also that it can be helpful in foreseeing the prognosis. So, it is an important marker in diagnosis, predicting mortality and the probability of septic shock with organ damage [6-7]. It is recommended that patients arriving at the emergency department or clinic with necrotizing fasciitis, cellulitis, or any soft tissue infections must be tested for PCT levels. It is unlikely that a patient with the etiological factors of NF, a supplementing history, and necrotic tissue are considered for PCT levels. Instead, a more invasive plan of tissue debridement and the microbiological evaluation of a sample is always preferred, which requires a surgical team, paraphernalia, and pathology technicians. It is likely that after receiving a debrided specimen, further culture is required, which needs more time.

However, with PCT levels, the extent of inflammation can be estimated. These levels can guide the physician about the underlying morbidity and help in early diagnosis, and empiric treatment initiation can lead to a significant reduction of tissue invasion by bacterial sepsis. It can be used both preoperatively and postoperatively in determining the diagnosis and prognosis of NF. In addition to PCT, the diagnosis can be fortified using LRINEC scoring.

LRINEC scoring was introduced by Wong et al. to differentiate NF from other soft tissue infections. Wong’s study illustrated that by using LRINEC scoring, NF can be diagnosed early, which can entirely change the course of therapy and affect the outcomes. This study was interpreted by using the regression coefficient of a predictive factor and multiple logistic regression models. Its positive predictive value was 92%, making this system a promising way of distinguishing necrotizing fasciitis from other necrotizing soft tissue infections (NSTIs) [8]. LRINEC scoring comprises C-reactive protein (CRP), white blood cell count, hemoglobin level, creatinine, sodium, and glucose. A score of six or more suggests a high probability of having necrotizing fasciitis and a score of less than six leads to lower chances of having NF. A high score gives us a temporal relationship with increased mortality, thus needing urgent treatment for a favorable prognosis.

Another study was conducted in Weil Cornell University, Qatar, in 2017, in which Wong`s model was used to test the efficacy of this scoring system. It is believed that this scoring for NF can predict hospital outcomes and find ill patients, providing them with a more patient-centered treatment. By using both PCT and LRINEC scoring, the diagnosis of NF can be established and adequate measures can be taken in the early course of the disease. In critical care, the timing of the clinical staff and the intensity of disease both contribute to the outcome of the patient’s health. Having a rapid laboratory test can reduce the time to diagnosis and impact morbidity. So, both LRINEC score and PCT can be used for the non-invasive diagnosis of necrotizing fasciitis and differentiate it from other soft tissue infections [2].

Other markers in diagnosing necrotizing fasciitis

While there is an emphasis on PCT and the LRINEC scoring system, increased levels of C-Reactive Proteins (CRP) and “pain out of proportion” can also be contributing factors to the clinical score in necrotizing fasciitis. A study suggested that there is a five-fold increase in CRP in necrotizing fasciitis [9]. CRP contributes to the LRINEC score and is related to PCT, considering both share an inflammatory origin. This study also suggested that 60% of the patients with necrotizing fasciitis presented with significant pain because, in cellulitis, there is mild to no pain. Apart from CRP and "pain out of proportion," there are many other co-morbidity factors contributing to the outcome of the disease. Some of these factors include hypertension, diabetes mellitus, and fat accumulation. These co-morbid factors and CRP (levels greater than 150 mg/liters), low Hb, low erythrocytes, and severe pain can all serve as major factors in determining the diagnosis of necrotizing fasciitis. Therefore, these markers should also be added to the current scoring system and a modified scoring system should be devised for better patient care [9]. Another study done in a multicenter setting in Belgian hospitals demonstrated that markers such as creatine kinase, CRP, and creatinine were higher in patients with necrotizing fasciitis then in cellulitis, so these markers can help in differentiating NF from another set of similar soft tissue infectious diseases with less intensity and superficiality. But, neither CRP nor creatine kinase had a prognostic value among the necrotizing fasciitis group and can only be isolated markers for diagnosis [10]. Another study showed that using a serum lactate value of >2.0mmol/l at presentation can be a useful parameter in determining tissue necrosis. It can adjunct the diagnosis in necrotizing fasciitis, which could help in speed up proper management [11].

Conflicts in Wong`s model

A study in California showed that LRINEC scoring did not differentiate between necrotizing fasciitis and cellulitis since the scores were high in cellulitis too [12]. It was also seen that CRP levels were considered an important contributor to the overall score, and the system worked more in diabetic populations than in the non-diabetic population. Also, a case presented by Wilson et al. showed that this system didn't diagnose necrotizing fasciitis in his patient, suggesting that this system can only lead to clinical suspicion rather than directing patient care [13]. This contradicts the earlier-mentioned Wong’s model and brings to us many questions regarding the system's efficacy, requiring more evidence of established facts. In one way, the clinical efficiency of this model with PCT levels can help in early diagnosis but reliance on these measures cannot be absolute. To some extent, they can help in early diagnosis, but Wilson’s suggestion is important and should be probed further with more extensive cohort models in multiple regions before considering it a norm. Most studies back this idea, which makes it a successful, non-invasive way of diagnosis, even with the reported conflicts.

Current practices and future considerations

LRINEC scoring and PCT are non-invasive methods by which we can detect necrotizing fasciitis earlier and reduce the need for surgical exploration. This can offer the patient an earlier treatment and a favorable prognosis. Current surgical and medical practices encourage an invasive diagnostic plan that involves tissue debridement and the evaluation of microorganisms. This is a time-consuming process and may involve logistics or workforce in multiple steps. The gold standard diagnostic tests for necrotizing fasciitis are surgical exploration and a microbiological and histopathological analysis of soft tissue [1]. However, because of the aforementioned reasons, this spreading disease can cause sepsis and can prove fatal. One important consideration is that the surgical debridement for NF is done in very strict aseptic measures that are not efficient in many settings of developing countries. Also, the extensive tissue lost can cause hypothermia, loss of tissue homeostasis, and multiple other complications. In a surgical setting, there is also an issue of anesthesia choice, which, in a large wound treatment, may need general anesthesia, but the comorbidities of the patient may not allow proceeding.

It is recommended that PCT and LRINEC scoring, along with similar non-invasive ways should be brought into clinical practice with associated measures of their outcomes by testing studies in various regions, ethnic groups, types of NF, and for different organisms. The efficacy of these non-invasive, bedside tests should be considered, and multiple studies should be encouraged to bring this quick method of diagnosing and, later, treating the illness into the major medical circuit, and more arguments should be known and discussed by clinicians and research physicians. For now, it is known that these methods, including comprehensive LRINEC scoring, can help in this disease. Modifications in the scoring system, the addition of parameters, and validation, along with the limitations, should all be considered.

The surgical debridement and evaluation of the specimens for the organisms is an absolute diagnosis but such non-invasive and quickly performed tests can aid in early diagnosis. The aim is to reduce the time from diagnosis to surgical intervention, if needed, to reduce the complications that may arise due to the progression of such similar conditions. The idea of practicing such scales, including the LRINEC scoring system, Fournier's gangrene severity index (FGSI), the Wagner ulcer grade classification, and many other such mathematical ways of making the diagnosis are to aid the process of diagnosis; the final diagnosis still in practice is the standardized technique. The LRINEC scoring system comes with a high predictive value, which suggests that such calculations can be done in a preoperative setting to know the status of the illness and can be used to comment on the course of morbidity. Also, radiological investigations are encouraged to know the extent of tissue invasion. It is important to emphasize that all such diagnostic techniques are towards achieving better patient care and assisting in reaching a better prognosis.

Fournier`s gangrene severity index and imaging

Apart from the non-invasive methods, it is important that we emphasize, in detail, that other diagnostic modalities (as mentioned above) have also been considered in the past, for example, Fournier`s gangrene severity index and imaging modalities. Fournier's gangrene (FG) is a fulminant infection, including necrotizing fasciitis of the genital, perineal, and/or perianal regions. Its clinical presentation is variable, but it often presents with edema, erythema, pain, fever, and increased volume; crepitus is present in 50%-62% of cases [14]. The time interval from the onset of symptoms specific to the process until the request for medical care is from two to seven days, on average. This time determines the extent of the necrotic area and is a critical influence on the prognosis [15]. The severity index of Fournier was described by Loar et al. and is used to stratify risk and prognosis. It is also a good tool for predicting the severity of the disease and the mortality risk of the patients. FGSI is a numerical score obtained from a combination of physiological hospital admission parameters that include temperature, heart rate, respiration rate, sodium, potassium, creatinine, leukocytes, hematocrit, and bicarbonate. A retrospective study conducted in 95 patients with Fournier`s gangrene demonstrated that FGSI > 9 is important for predicting severity factors in Fournier`s gangrene [16]. Another study conducted in 85 male patients with Fournier`s gangrene was done to compare the demographic information and the nine parameters of Fournier's gangrene severity index between survivors and non-survivors. They observed that in 16 non-survivors, FGSI was increased when compared to survivors and three of the parameters were changed, which included serum potassium, creatinine, and hematocrit; the rest of the parameters of FGSI were unchanged. They then concluded that a simplified form of FGSI can also be used to compare outcomes in two different populations [17]. Furthermore, imaging such as magnetic resonance imaging (MRI), computed tomography (CT), and ultrasound can also aid in the diagnosis of necrotizing fasciitis. Of these imaging modalities, MRI is superior to all of them and can help initiate appropriate management in a timely manner [18]. The role of other imaging modalities is limited. In radiography, the presence of soft-tissue gas is the only specific sign of necrotizing fasciitis but is seen only in a limited number of cases (e.g. in the presence of gas-forming organisms). Alternately, radiographs may show non-specific findings of soft-tissue swelling and edema. CT performs better than radiography and it has cross-sectional imaging capabilities that can reveal intermuscular fluid collections and fascial plane thickening with variable contrast enhancement. Lastly, ultrasonography is also useful, as it can demonstrate the presence of diffuse edema, a thickness of the scrotal wall and possibly the penis, and the presence of scrotal gas and, finally, an ultrasound can also aid in imaging-guided diagnostic fluid aspiration [14,18]. Even though all these clinical scales and tests (mentioned above) can aid in the diagnosis of necrotizing fasciitis, in equivocal cases, the definitive diagnosis is attained by exploratory surgery at the infected sites and should not be delayed [19-20].

## Conclusions

This study suggests that using non-invasive tests, such as PCT, the LRINEC scoring system, and other markers (CRP, creatine kinase, "pain out of proportion," creatinine, and lactate), can detect necrotizing fasciitis early in its course. The standard method comprises using a surgical intervention that is invasive and can be time-consuming. This method is basic and helps achieve earlier treatment, thus, providing a better prognosis.
